# Single‐Cell Patch‐Clamp/Proteomics of Human Alzheimer's Disease iPSC‐Derived Excitatory Neurons Versus Isogenic Wild‐Type Controls Suggests Novel Causation and Therapeutic Targets

**DOI:** 10.1002/advs.202400545

**Published:** 2024-05-21

**Authors:** Swagata Ghatak, Jolene K. Diedrich, Maria Talantova, Nivedita Bhadra, Henry Scott, Meetal Sharma, Matthew Albertolle, Nicholas J. Schork, John R. Yates, Stuart A. Lipton

**Affiliations:** ^1^ Neurodegeneration New Medicines Center The Scripps Research Institute La Jolla CA 92037 USA; ^2^ Department of Molecular Medicine The Scripps Research Institute La Jolla CA 92037 USA; ^3^ Quantitative Medicine and Systems Biology The Translational Genomics Research Institute Phoenix AZ 85004 USA; ^4^ Department of Neurosciences School of Medicine University of California, San Diego La Jolla CA 92093 USA; ^5^ Present address: School of Biological Sciences National Institute of Science Education and Research (NISER)‐Bhubaneswar, an OCC of Homi Bhabha National Institute Jatani Odisha 752050 India; ^6^ Present address: Drug Metabolism and Pharmacokinetics Department Takeda Development Center Americas San Diego CA 92121 USA

**Keywords:** Alzheimer's disease, hiPSC‐derived neurons, patch clamp electrophysiology, single‐cell proteomics

## Abstract

Standard single‐cell (sc) proteomics of disease states inferred from multicellular organs or organoids cannot currently be related to single‐cell physiology. Here, a scPatch‐Clamp/Proteomics platform is developed on single neurons generated from hiPSCs bearing an Alzheimer's disease (AD) genetic mutation and compares them to isogenic wild‐type controls. This approach provides both current and voltage electrophysiological data plus detailed proteomics information on single‐cells. With this new method, the authors are able to observe hyperelectrical activity in the AD hiPSC‐neurons, similar to that observed in the human AD brain, and correlate it to ≈1400 proteins detected at the single neuron level. Using linear regression and mediation analyses to explore the relationship between the abundance of individual proteins and the neuron's mutational and electrophysiological status, this approach yields new information on therapeutic targets in excitatory neurons not attainable by traditional methods. This combined patch‐proteomics technique creates a new proteogenetic‐therapeutic strategy to correlate genotypic alterations to physiology with protein expression in single‐cells.

## Graphical abstract

A single‐cell patch‐clamp/mass spectrometry‐based proteomics platform (scPatch‐Clamp/Proteomics) is developed to discover in diseased versus control neurons differentially‐expressed proteins as potential therapeutic targets. Human (h)iPSC‐derived excitatory neurons with Alzheimer's disease (AD) mutation or isogenic control are studied. Mediation analysis shows AD mutation drives protein changes, which then mediate a hyperelectrical phenotype in AD hiPSC‐neurons, similar to that observed in AD brain.

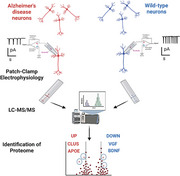

## 1 Introduction

**Figure 1:**
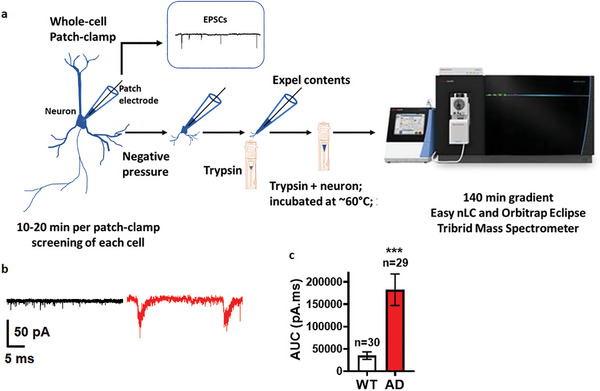
Schematic of data acquisition and analysis overview. a) Schematic of the workflow of scPatch‐Proteomics experiments. b) Representative whole‐cell recordings with patch electrodes under voltage‐clamp for excitatory post synaptic currents (EPSCs) from WT (black) and AD (red) hiPSC‐neurons. c) Quantification of area under the curve (AUC) from EPSC traces of 20 s epochs from WT and AD hiPSC‐neurons. Sample size listed above the bars. Data are mean ± SEM. Statistical significance analyzed by Student's t‐test.

Investigating the relationship in single excitatory neurons between aberrant protein expression and abnormal hyperexcitability observed in human Alzheimer's disease (AD) brains^[^ ^1^, ^2^, ^3^, ^4^, ^5^ ^]^ requires a novel approach for single‐cell analysis of the proteome as it relates to the hyperelectrical phenotype. Single‐cell proteomics has advanced rapidly for analyses of mammalian cells,^[^ ^6^, ^7^, ^8^, ^9^, ^10^, ^11^, ^12^, ^75^, ^76^ ^]^ consequently allowing us to develop a new single‐cell patch‐clamp/proteomics (liquid chromatography‐tandem mass spectrometry [LC‐MS/MS]) platform termed scPatch‐Proteomics, combined with bioinformatics mediation analysis, to overcome this technical limitation. With our improved techniques using human induced pluripotent stem cell (hiPSC)‐derived excitatory neurons, we were able to detect over 2250 proteins in a single neuron (**Figure** **1**a) compared to ≈275 proteins in prior studies.^[^ ^12^ ^]^ This improved yield of proteins over any prior single‐cell technique afforded us the power to conduct an unprecedented bioinformatic analysis on single neurons and correlate their protein expression to their electrical activity. Previously, we and others found a hyperexcitability phenotype in AD hiPSC‐derived cerebrocortical neurons (AD hiPSC‐neurons) bearing either amyloid precursor protein (APP) or presenilin 1 (PS1) patient mutations compared to isogenic, gene‐corrected wild‐type (WT) control neurons.^[^ ^13^, ^14^ ^]^ In fact, this excessive electrical activity resembled that seen in human AD brains on electroencephalograms (EEGs) in several respects,^[^ ^1^, ^2^, ^3^, ^4^, ^13^, ^14^ ^]^ suggesting that AD hiPSC‐neurons represent a model system for at least that aspect of the disease. Since the hyperexcitable phenotype has also been linked to synaptic damage,^[^ ^13^, ^14^ ^]^ which is the best neuropathological correlate to cognitive decline in AD,^[^ ^15^, ^16^ ^]^ these findings gave credence to the use of AD hiPSC‐neurons in modeling some aspects of the human AD brain.

The standard approach involving the comparison of bulk proteins in the AD brain to controls loses the specific cell‐type identity of these proteins. Moreover, more rare proteins would be obfuscated by this approach. Here, we can pick a single neuronal cell type, in our case excitatory (glutamatergic) neurons based on their electrophysiological characteristics and sample their proteomic differences between AD and isogenic WT while also monitoring their electrical activity. Initially, we looked at the differentially expressed proteins (DEPs) in AD compared to isogenic WT excitatory hiPSC‐neurons and found several important proteins already linked to AD pathogenesis and several other DEPs not previously linked to AD that might serve as novel therapeutic targets. To further this analysis, we next looked at the correlation between hyperelectrical activity observed in single AD hiPSC‐neurons under patch clamp and the DEPs found in AD hiPSC‐neurons. In this manner, we found additional DEPs in AD excitatory hiPSC‐neurons, potentially reflecting their hyperelectrical phenotype.

Here, we highlight correlative and mediation analyses of the proteins expressed abnormally in AD hiPSC‐neurons versus isogenic control as a predictor of their hyperelectrical phenotype, and thus with therapeutic potential. Our mediation analysis implies that these aberrantly‐expressed proteins likely contribute to the hyperexcitable phenotype. Finally, we present Reactome pathways in which DEPs found in AD hiPSC‐neurons are involved, thus suggesting possible future lines of exploration for AD pathogenesis.

Recently, single‐cell (sc) or single nuclear (sn)RNA‐seq has become routine in human brain or hiPSC‐derived cells in both healthy and diseased states. The transcriptome, however, does not always faithfully reflect the translated proteome, and therefore scRNA‐seq may not be sufficient for analysis of cell function, for example, neurons in the AD brain, at the level of explaining their phenotypic behavior such as hyperelectrical activity. Along these lines, a recent multi‐omics study showed that proteomic signatures in the postmortem AD brain do not match the transcriptomic signature in all cases.^[^ ^17^ ^]^ Therefore, our new approach to single‐cell patch‐proteomics with bioinformatics offers a platform for more detailed correlation and causal mediation analyses between protein expression and the physiological behavior of single cells.

## 2 Results

**Figure 2:**
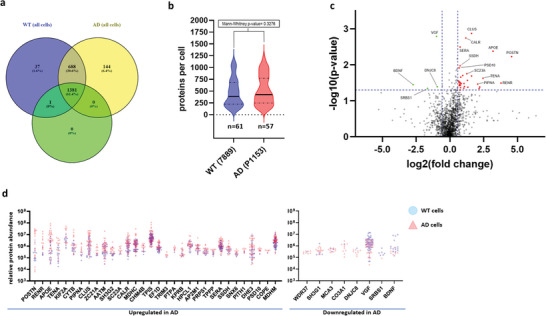
Initial scPatch‐Proteomic analysis. a) Proteins per cell for all hiPSC‐neurons analyzed (including proteins not passing the subsequent first‐pass exclusion criteria of being present in ≥5 cells and having ≥100 proteins). Venn diagram represents number of proteins detected from LC‐MS/MS data in WT hiPSC‐neurons (blue) and AD hiPSC‐neurons (yellow), totaling 2251 proteins. The proteins present in these cells after first‐pass exclusion are shown in the green circle; in this case 1382 proteins were detected in either or both the WT and AD hiPSC‐neurons. b) Proteins per cell after first‐pass exclusion used in all subsequent analyses. Thick line denotes median value, dashed lines denote quartiles. c) Volcano plot for proteins analyzed with cut‐offs for significance marked by blue dashed lines, representing ≥1.5x FC with *P* < 0.05 (AD proteins relative to WT, with green dots signifying proteins that are less abundant in AD hiPSC‐neurons relative to WT, and red dots, proteins that are more abundant in AD hiPSC‐neurons than WT). In this analysis, 32 proteins were found to be significantly upregulated in AD hiPSC‐neurons and 4 downregulated relative to WT. d) Overlaid linearized scatterplot for relative protein abundance (by label‐free quantification); blue circles represent values for WT hiPSC‐neurons and red triangles, AD hiPSC neurons. Note there are a number of values of 0, indicating that these proteins were not detected in every cell.

In our platform for single‐cell proteomics and whole‐cell recording from AD hiPSC‐neurons or WT isogenic controls, we aspirate and lift the whole neuron with the patch electrode for transfer directly to an LC/MS autosampler tube for mass spectrometry analysis of the cell's proteome (Figure 1a). In this study, we compared AD mutant hiPSC‐neurons expressing heterozygous presenilin 1 (PSEN)1‐mutant (M146V/WT) to an associated isogenic (WT/WT) control.^[^ ^18^, ^19^ ^]^ We successfully collected data on 140 excitatory hiPSC‐neurons (AD = 57 and WT = 73), and were able to detect 2251 different proteins in these cells (Table S1, Supporting Information). The distribution of these proteins in AD and WT hiPSC‐neurons is shown in a Venn diagram (**Figure** **2**a). Of these recordings, 138 (WT = 73 and AD = 55) manifested spontaneous action potentials under current clamp and/or excitatory postsynaptic currents under voltage clamp during recording with a patch electrode. Assessable proteomics data, defined as each cell to be analyzed containing at least 100 identifiable proteins plus each protein being present in at least 5 cells by LC‐MS/MS (see Experimental Section), were successfully obtained on 118 of these hiPSC‐neurons (WT = 61 and AD = 57). With this filter, we were able to detect 1382 proteins from single hiPSC‐neurons (Figure 2a,b; Table S2, Supporting Information, all data sheet 1). Considering the 118 hiPSC‐neurons from which we obtained assessable proteomics data, Figure 2c shows a Volcano plot of statistical probability versus log_2_(magnitude of change or fold change [FC]) of proteins found in AD versus WT hiPSC‐neurons. Violin plots (Figure 2b) and an overlaid linearized scatterplot (Figure 2d) are shown for the proteins up or downregulated by label‐free quantification in AD versus WT hiPSC‐neurons.

In this analysis, we initially examined the relationship between mutant status (AD versus isogenic WT) and detected protein abundance (Figure 2c). We note that we did not observe outliers or problematic distributions of the protein abundances and electrophysiological data that might have confounded inferences based on simple linear regression analyses. The protein that was statistically most upregulated in the AD hiPSC‐neurons compared to WT control was **CLU** (clusterin or CLUS), which functions as an extracellular chaperone contributing to lipid transport and immune modulation, and has been previously implicated in AD pathogenesis, potentially by affecting amyloid‐β (Aβ) peptide aggregation or clearance.^[^ ^20^ ^]^ The next most upregulated protein in AD excitatory hiPSC‐neurons compared to WT was **CALR** (calreticulin), important in inflammatory NF‐κB signaling, and binding to Ca^2+^ and misfolded proteins to prevent them from being transferred from the endoplasmic reticulum (ER) to the Golgi apparatus. Interestingly, this protein had been previously thought to be downregulated in postmortem AD brain.^[^ ^21^, ^22^ ^]^ Since our hiPSC‐neurons represent an early stage of development of AD, it is possible that the increase represents initial compensation for the disease process, which later decompensates.

**APOE** was also upregulated, and is well‐known for its involvement in AD, as discussed further below. Another upregulated protein in AD hiPSC‐neurons was **POSTN** (periostin), important in cell attachment and spreading, but transcripts of the encoding gene were reportedly downregulated in late‐stage postmortem AD brain.^[^ ^23^ ^]^ Again, the concept of initial compensation or counterresponse in early stages of the disease remains possible.

**SSDH**, or mitochondrial NAD^+^‐dependent succinic semialdehyde dehydrogenase, was upregulated and represents a key enzyme in the tricarboxylic acid (TCA) cycle, converting succinic semialdehyde (SSA) into succinate, and thus important for energy metabolism in neurons. To our knowledge, its elevation has not been previously reported in AD brain. SSDH is also important for the GABA shunt, an alternative to the TCA cycle, but in this case, SSDH deficiency results in an increase in the inhibitory transmitter GABA.^[^ ^24^ ^]^ Metabolic therapies for AD have recently been suggested, but SSDH represents a novel target in the TCA cycle not previously proposed, as discussed further below.

Another upregulated protein, SERA (O43175, or D‐3‐phosphoglycerate dehydrogenase or PDGDH), catalyzes reversible oxidation of 3‐phospho‐D‐glycerate to 3‐phosphonooxypyruvate, the first step of the phosphorylated l‐serine biosynthesis pathway, 2‐hydroxyglutarate to 2‐oxoglutarate, or (S)‐malate to oxaloacetate in the TCA cycle. This enzyme was found to be increased in AD brain,^[^ ^25^ ^]^ but this report has been contested.^[^ ^26^ ^]^ Our finding that it is upregulated specifically in excitatory neurons may explain prior discordant results using different brain samples composed of disparate cell types.

In contrast to these upregulated proteins, downregulated proteins in AD hiPSC‐neurons compared to isogenic WT include **VGF** (VGF nerve growth factor inducible factor) and **BDNF** (brain‐derived neurotrophic factor). VGF and BDNF are known to be neuroprotective neurotrophic factor pathways that are downregulated in postmortem human AD brains and thus have been implicated in AD pathogenesis.^[^ ^27^, ^28^ ^]^ The next most downregulated protein in the AD hiPSC‐neurons was **DNJC8** (or heat shock chaperone HSC70), whose modulation has been proposed as a therapeutic for AD because chaperones can be used to refold aggregated proteins.^[^ ^29^ ^]^ However, to our knowledge, this is the first report of specific downregulation of a chaperone in excitatory neurons in AD. **SRBS1** (or CAP/Poinsin protein, a.k.a. Sorbin, an SH3 domain‐containing protein) was also significantly downregulated. This protein, encoded by the gene *SORBS1*, is an adaptor protein regulating cell adhesion and growth factor signaling. Expression of this and related genes are reportedly downregulated in the human AD brain due to hypermethylation.^[^ ^30^ ^]^

Notably, considering the DEPs determined from all 118 cells (for AD vs WT hiPSC‐neurons), gene ontology (GO) profiler intersection analysis (Table S3, Supporting Information) showed several of the altered proteins were involved in related pathways, for example, the highest‐ranking pathway of APOE, CALR, PTPA, AP2M1, SNX6, VGF, and BDNF. In this gene set enrichment analysis (GSEA), only enriched pathways are highlighted in the list.

### 2.1 Linear Regression Analysis of the Relationship of Mutant AD Status and Protein Abundance in Single AD hiPSC‐Neurons Versus Isogenic WT

**Figure 3:**
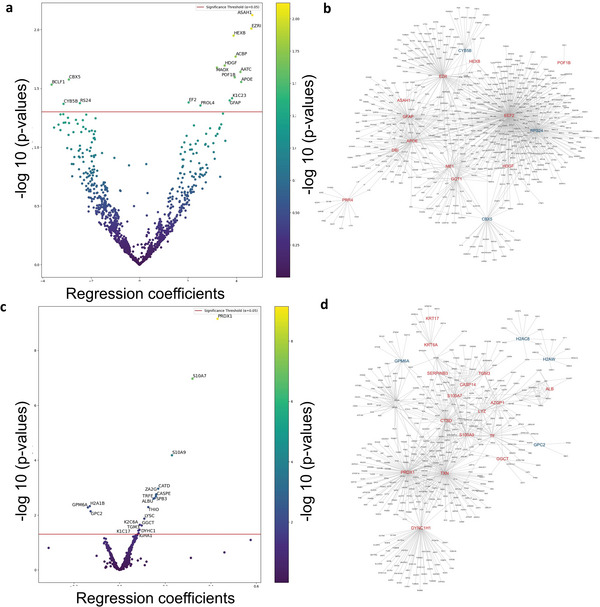
Linear regression analysis. a) Volcano plot of linear regression model results with protein abundance taken as the dependent variable and AD mutant status of hiPSC‐excitatory neurons taken as the independent variable. By this analysis, 13 proteins were upregulated, and 4 proteins were downregulated in AD hiPSC‐neurons compared to isogenic WT; redline indicates protein FC ≥ 1.5 and *P* < 0.05 (full dataset in Table S5, Supporting Information, sheet for Model 1). b) STRING Reactome pathway analysis of significantly up and downregulated DEPs from panel a. c) Volcano plot of linear regression model results with log(AUC) of hyperelectrical activity taken as the dependent variable (or effect/outcome) and protein abundance and AD mutant status of hiPSC‐excitatory neurons taken as the independent variables (or cause). Under this analysis, 17 proteins were upregulated, and 3 proteins were downregulated; redline indicates protein FC ≥ 1.5 and *P* < 0.05 (full dataset in Table S5, Supporting Information, sheet for Model 3). d) STRING Reactome pathway analysis of significantly up and downregulated DEPs from panel c.

Next, we wanted to examine the relationship between mutant status (AD vs isogenic WT), detected protein abundance, and the hyperelectrical activity phenotype seen in AD excitatory neurons in a more stringent and robust fashion, possibly allowing us to detect proteins statistically related to AD not seen with standard methods.^[^ ^31^ ^]^ For this analysis, we had 57 of the recorded hiPSC‐neurons (WT = 28 and AD = 29) that had full datasets for both protein analysis and electrophysiological assessments, as determined in a masked fashion with the observer blinded to genotype (see Table S4, Supporting Information for quantification of proteins present in these cells). Note that this is in contrast to the analysis reflected in Figure 2c, which considered all 118 of the cells analyzed by proteomics, even if they did not have adequate electrical measurements for this further analysis. On the 57‐cell subset of the data, we first performed a linear regression analysis (see Experimental Section) to test the association of protein abundance and AD mutation status without consideration of electrical properties. Thus, this linear regression analysis can be used to test the association between protein abundance and AD mutational status directly without initial consideration of the hyperexcitable phenotype. This approach mathematically models the protein abundance (the dependent variable) as a linear function of mutation status (the independent variable). Note that by using this linear regression normalization method, we were able to compare these results to subsequent mediation analysis of the events to predict causation, as discussed below. The Volcano plot in **Figure** **3**a (dataset in Table S5, Supporting Information, sheet for Model 1) shows the log_10_(*P*‐value) for association against the normalized regression coefficient. The plot relates AD mutation as the independent variable (or cause) to protein abundance as the dependent variable (or effect/outcome) in hiPSC‐neurons. In this analysis, we found 13 proteins were significantly upregulated, while 4 proteins were downregulated after setting statistical significance at *P* < 0.05. As expected, several of the proteins detected in this manner were in related pathways or identical to those shown in the larger dataset in Figure 2c, such as APOE.

In the linear regression analysis (Figure 3a), the top hit among upregulated proteins, **ASAH1** (or N‐acylsphingosine amidohydrolase 1), is known to promote senescent cell survival.^[^ ^32^ ^]^ This protein functions as an acid ceramidase, which cleaves fatty acids from ceramide, generating sphingosine (SPH). In turn, SPH is phosphorylated by sphingosine kinase to form sphingosine‐1‐phosphate (S1P), whereas ceramide itself has been linked to apoptosis in AD. ASAH1, therefore, may possibly represent a counterresponse to the disease process that protects neurons in the early stages of the disease, as represented here in these AD hiPSC‐neurons. Recently, cell senescence has been proposed to be important in AD, so we posit that ASAH1 may represent a unique target for future drug therapy.

Interestingly, another top‐upregulated protein is **HEXB**, the β subunit of β‐hexosaminidase A and β‐hexosaminidase B, whose deficiency is known in childhood disease, but in adulthood is also important, located within lysosomes, where the enzymes break down sphingolipids. The fact that two of the top 3 upregulated proteins are involved in sphingolipid metabolism could signal an important involvement of these lipids in AD, as has been suggested previously.^[^ ^33^ ^]^

The second‐most upregulated protein, **EZR1**, is important in cell adhesion and may foster proliferation of certain types of cancers.^[^ ^34^ ^]^ Its potential role in AD was previously unknown and could represent an interesting therapeutic target for future exploration. In contrast, another highly upregulated protein on the list is **APOE** (similar to our finding in Figure 2c), known to be involved in AD pathogenesis, and thus serves as a control hit in some sense. In fact, APOE is known to be upregulated by neurons under stress conditions and aging, and neuronal APOE has been reported to be associated with increased excitability in the context of AD.^[^ ^35^ ^]^

An unusual and perhaps surprising case of upregulation in AD hiPSC‐neurons compared to isogenic WT is that of **GFAP** (glial fibrillary associated protein). In addition to being an astrocyte marker, however, GFAP is also found in radial glial cells, a neural stem/precursor cell (NPC) that gives rise to excitatory neurons.^[^ ^36^ ^]^ Thus, it is possible that although these cells exhibited characteristic neuronal electrical activity (narrow time‐based action potentials, excitatory postsynaptic currents, etc.), the AD mutant genotype increased or prolonged the neural progenitor stage, as has been suggested for PSEN1 mutation because of its effect on NOTCH signaling;^[^ ^37^ ^]^ alternatively, the AD mutation could be associated with de‐differentiation back toward the NPC stage. Contamination of the protein analysis from another cell type is unlikely since the neurons were not plated on astrocytes and displayed multiple neuronal markers, and all cells recorded from in these cultures manifested electrical properties of neurons.

The next most upregulated protein in the AD hiPSC‐excitatory neurons was **ACBP** (also known as **DBI**), representing Acyl‐CoA‐binding protein, a small (10 kDa) protein that binds acyl‐CoA esters affecting lipid (fatty acid) metabolism.^[^ ^38^ ^]^ These derivatives can be degraded in mitochondria to acetyl‐CoA to fuel the TCA cycle. TCA cycle dysfunction has been reported in human neurons in AD and other related dementias; this dysfunction contributes to energy compromise, with consequent synaptic damage.^[^ ^39^, ^40^ ^]^ Since synapse loss is a close neuropathological correlate to cognitive decline in AD,^[^ ^15^, ^16^ ^]^ this finding has disease‐modifying implications. Moreover, ACBP may also interact with the benzodiazepine binding site of GABA_A_ receptors to affect inhibitory neurotransmission, which could contribute to hyperexcitability. ACBP has been reported to be increased in the cerebrospinal fluid (CSF) of AD patients, and inhibition of ACBP may increase autophagic flux and thus clearance of misfolded proteins.^[^ ^41^ ^]^ Thus, targeting ACBP in excitatory neurons could represent a novel approach to AD therapeutics.

**HDGF** (hepatoma‐derived growth factor), a heparin‐binding glycoprotein, was the next most upregulated protein in abundance in excitatory AD hiPSC‐neurons. It is known to signal via stimulating multiple pathways, such as MAPK and/or PI3K, and increasing the production of growth factors, including vascular endothelial growth factor (VEGF) that may possess anti‐apoptotic properties.^[^ ^42^ ^]^ Intriguingly, it has been reported to be increased in synaptic fraction of human brains from frail patients as they became cognitively impaired but not in patients with the diagnosis of AD.^[^ ^43^ ^]^ Thus, the increase in HDGF in our hiPSC‐neurons early in development of AD may represent a counterresponse, which if enhanced could be beneficial.

Among the most downregulated proteins, **CBX5** (chromobox protein homolog 5) represents protein HP1α, primarily functioning as a gene silencer. This action is dependent on interactions between the CD (N‐terminal chromodomain) and a methyl H3K9 mark. CBX5 activity is critical for normal regulation of genome expression and stability. In fact, this regulation has been shown to be disrupted with loss of Tau function in AD neurons,^[^ ^44^ ^]^ and thus CBX5 in excitatory neurons could represent a potential target for future therapy.

Also downregulated is **BCLF1** (or BCLAF1 protein, **Bcl‐2‐associated transcription factor 1),** which is involved in apoptosis, autophagy, and transcriptional control, as previously identified in tumorigenesis models.^[^ ^45^ ^]^ However, enhancement of its activity has been suggested to improve neurological dysfunction in the context of AD model systems.^[^ ^46^ ^]^ Thus, BCLF1 may also represent a novel therapeutic target in AD excitatory neurons.

A GO profiler analysis of the significant DEPs in Figure 3a is presented in **Table** **1**, and shows the involvement of signaling pathways that include sphingosine and lipid metabolism, synaptic plasticity and long‐term potentiation, autophagy, glial cell differentiation, and APOE in inflammation, consistent with the analysis of the individual DEPs presented above. Moreover, STRING Reactome analysis of the proteins affected in mutant AD hiPSC‐neurons differentially from WT to explore possible interactions (Figure 3b) showed network clusters for each protein and the relationship between them, suggesting new possible pathways to target therapeutically. In particular, interactions between ASAH1/sphingosine metabolism, GFAP/cytoskeletal proteins, APOE and ACBP (DBI)/fatty acid metabolism or GABA_A_ inhibitory receptors were noted.

**Table 1:** Pathway enrichment analysis of significantly dysregulated proteins in AD mutant hiPSC‐neurons compared to WT from Model 1 using g: Profiler. The Manhattan plot represents functional terms grouped and color‐coded by data source on the abscissa, and the corresponding enrichment *P*‐values in negative log_10_ scale on the ordinate. Each circle on the plot illustrates a single functional term. Various data sources are size‐scaled in accordance with the number of annotated genes in the specific term.

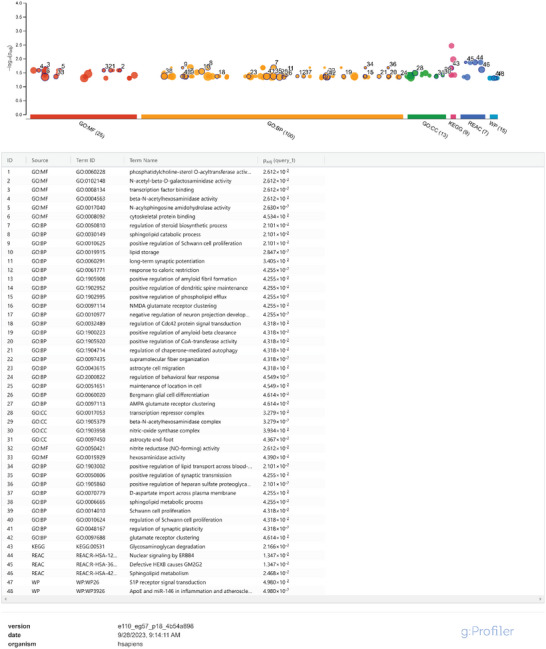

### 2.2 Linear Regression Analysis of the Relationship of Hyperelectrical Activity and Mutant AD Status to Protein Abundance in Single AD hiPSC‐Neurons Versus Isogenic WT

We had previously shown that AD hiPSC‐neurons manifest excessive spontaneous electrical activity,^[^ ^13^, ^14^ ^]^ similar to that seen in the intact human AD brain on EEG,^[^ ^1^, ^2^, ^3^, ^4^ ^]^ and additional recent studies also indicate that human AD brain manifests a hyperactive state and an excitatory transcriptomic signature early in the disease process.^[^ ^47^ ^]^

While we made measurements of many electrophysiological and membrane properties of both the AD hiPSC‐neurons and isogenic WT neurons (see Table S2, Supporting Information), we decided to use an area‐under‐the‐curve (AUC) analysis of excitatory currents to assess hyperelectrical activity under voltage clamp in each neuron during patch‐clamp recording. We chose this AUC because other parameters, for example, spontaneous action potentials (APs) measured during current‐clamp recording, could depend on the resting membrane potential (RMP) and this could be variable from cell to cell. In contrast, membrane potential could be tightly controlled during voltage clamp, under which current‐based parameters are analyzed, and thus considered to be more reliable. The major parameter of excitation recorded in voltage‐clamp was excitatory postsynaptic currents (EPSCs). However, to avoid the effect of superimposition of multiple EPSCs during voltage–voltage clamp recording obfuscating the determination of the exact number of EPSCs, we used AUC to sum the currents of these EPSCs during an epoch of 20 s of stable recording, and each of 57 hiPSC‐neurons (WT = 28 and AD = 29) met these criteria (Table S4, Supporting Information).

Figure 3c (based on data from the dataset in Table S5, Supporting Information, sheet for Model 3) shows a Volcano plot using linear regression analysis to relate log(AUC) of hyper electrical activity taken as the dependent variable (or effect/outcome) versus protein abundance and AD mutant status of hiPSC‐excitatory neurons taken as the independent variables (or cause). Considering AD mutation status, 17 proteins were positively associated with log(AUC) and 3 proteins were negatively associated with log(AUC) (*P* < 0.05 level shown by redline), indicating that they may be associated with the hyperactive phenotype. Proteins affected are important in redox regulation (upregulated in AD hiPSC‐neurons) and cell adhesion (downregulated in AD hiPSC‐neurons).

For example, among the significantly upregulated proteins were the following:

1. PRDX1 (peroxiredoxin 1)—a redox protein that can be reactive to protect neurons from nitro oxidative stress, as occurs in the AD brain. Under normal conditions, it is usually very low or absent in neurons in the brain,^[^ ^48^ ^]^ indicating that our result may reflect a reactive, early protective response to the disease. In fact, in human AD brain, PRDX1 has been reported to be elevated in some brain regions, including temporal cortex (reviewed in ref. [48]), but the exact cell type displaying the increase in PRDX1 (excitatory neurons in this case) was previously unknown and could represent a target worthy of further consideration.
2. THIO (thioredoxin 1), another redox regulator, has been reported to be decreased in an advanced stage, postmortem AD brain.^[^ ^49^ ^]^ Here, early in diseased excitatory neurons, its upregulation may again represent a protective response and a potential therapeutic target.
3. S10A7 and S10A9 (aka S100‐A7 and S100‐A9 proteins) were the next most‐upregulated proteins. S100 proteins are known to be increased in AD brain, often associated with protein inclusions. They are ligands for Receptor for Advanced Glycation Endproducts (RAGE) and are thought to participate in pro‐inflammatory responses.^[^ ^50^, ^51^ ^]^ These proteins have been proposed to form neurotoxic linear and annular amyloids, resembling Aβ protofilaments, and thus may represent a novel target in this regard.
4. CATD (Cathepsin D) was also upregulated. It is known to play a role in lysosomal function and autophagy in the AD brain to process amyloid precursor protein (APP) and Tau, and has been linked genetically to AD.^[^ ^52^ ^]^ To our knowledge, this is the first report of an increase in Cathepsin D in human excitatory neurons in AD, and may thus represent a new potential target in these neurons.

Proteins that were significantly downregulated include:

1. H2A3 (Histone H2A type 3), in agreement with histone modifications known to occur in human AD brain.^[^ ^53^ ^]^ The fact that this downregulation occurs in human excitatory AD neurons, however, was not previously known and could be important for disease pathogenesis.
2. GPM6A (glycoprotein M6A), a neuronal surface glycoprotein that is thought to facilitate calcium channel activity, thus promoting spine filopodia, dendrite, and synapse formation. Genetic alterations in the gene encoding GPM6A have been previously linked to AD and other human neurologic diseases,^[^ ^54^ ^]^ but dysregulation of the protein in human AD excitatory neurons was previously unknown and thus could represent a new therapeutic target.
3. GPCis2 (glipican2), an extracellular matrix (ECM) protein functioning as a cell adhesion molecule. Other family members have been implicated to be involved in human AD,^[^ ^55^ ^]^ but the finding of this additional family member could represent a novel target for drug development.

A GO profiler analysis of the significant DEPs in Figure 3c is presented in **Table** **2** and reveals signaling pathways involving inflammatory receptors, iron and zinc chaperone activity, redox‐active proteins, and transnitrosylation. Additionally, STRING Reactome analysis of the proteins affected in mutant AD hiPSC‐neurons differentially from WT to explore interactions (Figure 3d) shows potential interactions between redox‐active proteins, inflammatory pathways, and autophagy mediators, with additional connections to epigenomic regulators and dynein/microtubule transport. These findings have implications for AD pathogenesis.

**Table 2:** Pathway enrichment analysis of significantly dysregulated proteins in AD mutant hiPSC‐neurons compared to WT from Model 3 using g: Profiler. The Manhattan plot represents functional terms grouped and color‐coded by data source on the abscissa, and the corresponding enrichment *P*‐values in negative log_10_ scale on the ordinate. Each circle on the plot illustrates a single functional term. Various data sources are size‐scaled in accordance with the number of annotated genes in the specific term.

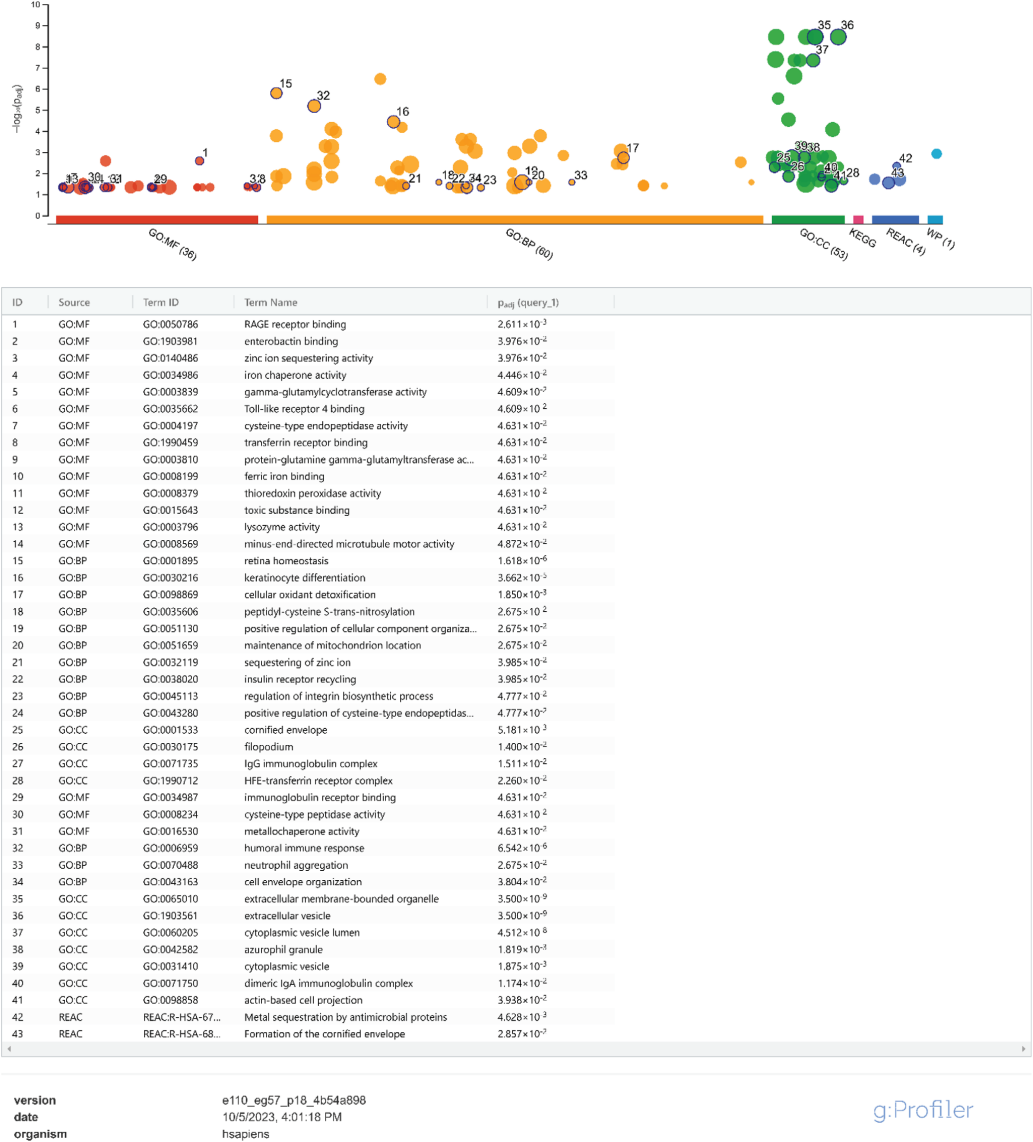

It is interesting to note that by linear regression analysis, the log(AUC) alone (without mutational status being considered) correlates with protein abundance in a manner similar to AD mutational status, with a very similar Volcano plot (Figure S1 from dataset in Table S5, Supporting Information, sheet for Model 2). Therefore, AD mutational status is associated with log(AUC), as might have been predicted from our prior studies linking AD mutation status to hyperelectrical activity.^[^ ^13^, ^14^ ^]^ Alternatively, this finding could suggest that alterations in DEPs caused by the AD mutation, in turn, contribute to the hyperexcitable phenotype. Therefore, we explored this possibility further below using mediation analysis.

### 2.3 Linear Regression‐Based Mediation Analysis to Predict Causation

**Figure 4:**
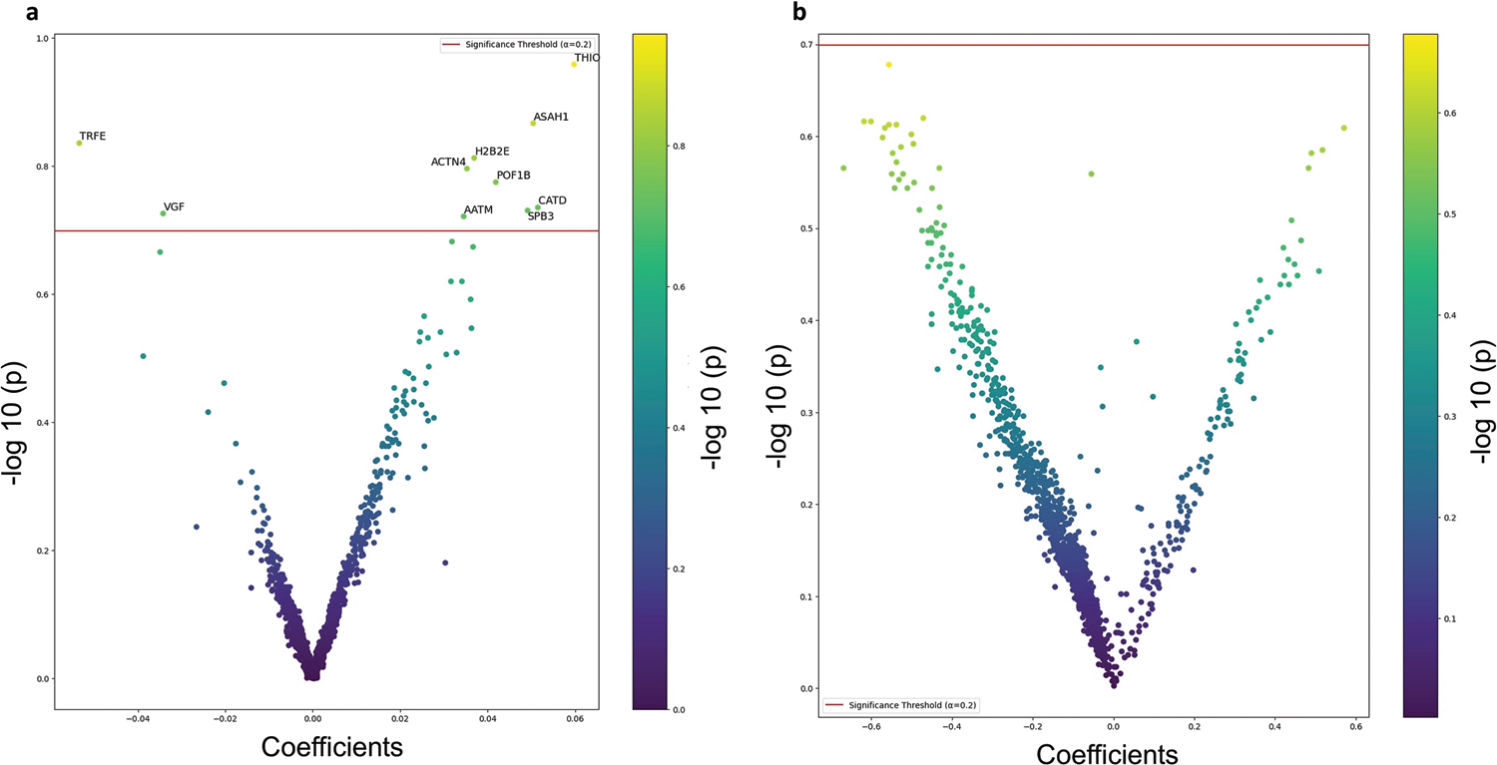
Linear regression‐based mediation analysis. a) Volcano plot of linear regression‐based mediation model with log(AUC) as the outcome, protein abundance as the mediator, and AD mutation status as the treatment that induces the change in protein abundance. In this analysis, 8 proteins were upregulated, and 2 proteins were downregulated. Red line represents log_10_(0.2) = 0.69 as an exploratory analysis, with 8 proteins upregulated and 2 proteins downregulated at that level (full dataset in Table S5, Supporting Information, sheet for Model 4). b) Volcano plot of linear regression‐based mediation model with protein abundance as the outcome, log(AUC) as the mediator, and mutation status as the treatment. Red line represents log_10_(0.2) = 0.69 as an exploratory analysis (full dataset in Table S5, Supporting Information, sheet for Model 5).

We pursued mediation analysis to further probe the causal relationship among AD mutational status, DEPs, and hyperexcitability of hiPSC‐neurons (see Experimental Section). **Figure** **4**a shows a Volcano plot employing a linear regression‐mediation model with protein abundance as the mediator, AD mutant status as the treatment, and hyperelectrical activity (AUC) as the outcome. Here, because of the hypothesis‐generating and exploratory nature of the analysis, we used a less stringent exploratory approach to see what pathways might possibly be affected by setting the analysis level at −log_10_(0.2) = 0.69 (or *P* < 0.2).

In this analysis, we identified 8 upregulated proteins as candidate mediators contributing to the hyperactivity phenotype (Figure 4a). The most upregulated protein was **THIO** (thioredoxin 1), reflecting redox alterations known to exist in human AD brain,^[^ ^56^ ^]^ and similar to the findings of the simple linear regression analysis (Figure 3c, above). Also similar to the initial regression analysis that confined attention to the relationship between protein abundance and log(AUC), the next most upregulated protein in the mediation analysis was **ASAH1** (N‐acylsphingosine amidohydrolase 1; compare Figure 4a to Figure 3a). Following this was **H2E2B** (**Histone H2B type 2‐E**), representing another histone protein and resembling that found in the simple linear regression analysis in Figure 3c. This histone is known to play a role in nucleosome remodeling, and various such histone modifications are known to occur in AD brain,^[^ ^53^ ^]^ as referred to above. Also upregulated was **ACTN4 (**α‐actinin‐4), an F‐actin crosslinking protein, which interestingly has been found to be associated with cognitive resilience in older individuals,^[^ ^57^ ^]^ raising the possibility that in this context in AD‐hiPSC neurons, it might represent an early counterresponse to AD pathology. Additionally, POF1B (premature ovarian failure 1B), another actin‐binding protein was found to be increased, as was CATD (cathepsin D), similar to the findings in the simple linear regression analysis shown in Figure 3c. Finally, SPB3 (Serpin B3), and AATM (mitochondrial aspartate aminotransferase) were also increased. SPB3 is a putative cysteine protease inhibitor, and other members of the serpin family have been associated with A**β** accumulation in AD brain.^[^ ^58^ ^]^ AATM, classically known as a liver enzyme, has also been reported to be increased in the CSF of Alzheimer's patients.^[^ ^59^ ^]^ To our knowledge, however, this is the first report of increased levels of these proteins in excitatory human AD neurons and therefore may point to pathogenic pathways in these neurons.

Considering downregulated proteins, there are two as potential mediators contributing to the hyperactivity phenotype:

**TFRE** (serum iron (Fe^3+^) transport protein transferrin (a.k.a. TF)). Note that iron dysregulation has previously been implicated in human AD brain, for example, with a decreased transferrin/iron ratio found in the basal ganglia.^[^ ^60^ ^]^ In this case, with transferrin being downregulated, this may reflect the known increase in iron‐dependent ferroptosis in neuronal cell death in AD. Recently, transferrin has been shown to play a protective role (at least in the liver) by preventing ferroptosis via iron binding,^[^ ^61^ ^]^ so the lower transferrin levels observed in AD hiPSC‐neurons compared to WT could contribute to neuronal damage. Interestingly, higher plasma transferrin levels in AD patients have been reported to be associated with more rapid cognitive decline, but the reasons for this remain unclear.^[^ ^62^ ^]^ Since this is the first report of TFRE dysregulation in human AD neurons, this finding may have both pathogenic and therapeutic implications.
**VGF** (VGF nerve growth factor inducible factor) was also downregulated, as found also in Figure 2c, above. VGF has also been reported to be decreased in human AD brain.^[^ ^63^ ^]^ VGF is a secreted protein thought to be important in energy homeostasis, metabolism, and synaptic plasticity. Since these processes are critically affected in AD brains, VGF may represent a potential avenue for future therapeutic exploration.

Collectively, with these aberrant protein changes as the mediator, AD mutant status as the treatment, and hyperelectrical activity (log(AUC)) as the outcome, this exploratory mediation analysis implies that AD mutation drives these protein changes, which in turn mediate the increased electrical activity.

To complement the analyses considering proteins as mediators of the relationship between mutant status and log(AUC), we also considered log(AUC) as the mediator of the relationship of mutant status and protein expression (i.e., differences in log(AUC) induced by mutant status affect protein abundance as a consequence of hyperelectrical activity). A volcano plot depicting these results is provided in Figure 4b. In this case, however, no protein associations were found. The implication of this finding for potential causal relationships between the mutant status, protein expression, and log(AUC) is that the aberrantly regulated proteins in AD mutant cells are not driven or mediated by the electrical hyperactivity directly, at least under these conditions.

## 3 Discussion

We have shown that scPatch‐Proteomics, coupled with specific data analysis techniques represents a feasible new platform for the study of the relationship of protein expression to disease‐inducing mutation at the level of the single neuron. This tour‐de‐force approach led to several reductionist observations not previously available to bulk proteomics on multiple cell types. For example, by exploring AD hiPSC‐neurons known for their hyperexcitable phenotype resembling that observed on EEG of human AD patients, we were able to correlate this aberrant electrical activity with AD genotype and DEPs using linear regression analysis. The analysis showed the relationship between several up and downregulated proteins known to be associated with AD and several not previously known, which may in fact represent new drug targets. Next, we performed an exploratory mediation analysis to probe the relationship among AD genotype, DEPs, and hyperexcitability further. By this analysis, we found that the main drivers of the aberrant electrical activity in AD hiPSC‐neurons were proteins involved in redox modulation; neuroinflammation; lysosomal function and autophagy; lipid, amino‐acid, and energy/carbohydrate metabolism (TCA cycle/mitochondrial function); iron transport and ferroptosis cell death; epigenetic regulation; and cell adhesion/cytoskeletal control of synaptic plasticity. These pathways complement and extend those recently reported for human AD brains by comprehensive snRNA‐seq, snATAC‐seq, and epigenomic studies.^[^ ^47^, ^64^, ^65^, ^66^, ^67^ ^]^

Moreover, when we compared our dataset to that of bulk human AD brain proteomics, we found reasonable concordance, the advantage with our dataset being that we could now identify the exact cell type that manifested changes in gene expression. For example, the integrated dataset of 7 human AD brain proteomes presented by Bai et al.^[^ ^77^ ^]^ largely agree with our results. Based on statistics performed by those authors, several proteins, including CLU, CALR, APOE, POSTN, HEXB, DB1, GFAP, HDGF, PRDX1, S10A9, CTSD, and SNX6, were all upregulated in AD, while VGF, BDNF, and GPM6A were downregulated, similar to our results on single excitatory human AD hiPSC‐neurons.^[^ ^77^ ^]^ Another study by Johnson et al.,^[^ ^78^ ^]^ which analyzed human AD brains and CSF samples, indicated that GFAP, CLU, APOE, DBI, CALR, PRDX1, CTSD, and AATM/GOT2 were upregulated, while VGF and GPM6A were downregulated,^[^ ^78^ ^]^ again reminiscent of our findings on single excitatory human AD hiPSC‐neurons. However, both of these studies showed that the expression levels of some proteins, namely BCLF1 and ACTN4, manifested opposite trends to what we observed.^[^ ^77^, ^78^ ^]^ Nonetheless, this difference might be attributed to our study on individual excitatory neurons, whereas these prior studies used bulk proteomics including all brain cell types.

Further along these lines, in agreement with our results, a quantitative proteomics study performed on prefrontal cortical samples of human AD brains showed that the proteins CTSD, DB1, ASAH1, and TPPP had enhanced expression in AD compared to controls, while SORBS1 was downregulated.^[^ ^79^ ^]^ Additionally, a neuropeptidomics study on AD human brain cortical synaptosomes, representing mainly excitatory neuronal endings, compared to age‐matched controls by Podvin et al.^[^ ^80^ ^]^ found a significant loss of VGF, which also aligns with our results in single AD hiPSC‐neurons. However, several DEPs that appeared on our list were not found in the DEP list of the above referenced studies.^[^ ^77^, ^78^, ^79^, ^80^ ^]^ These differences could arise because of the early stage of the disease at which the proteome was analyzed with our hiPSC‐based AD models compared to postmortem studies on AD brain, or because of the cell type(s) involved. Additionally, there were differences in the proteins identified among the various prior studies discussed above, indicating variability among samples and methods. Given this variability, the striking concordance of these previous findings and our own indicate the robustness of our dataset and support the conclusion that our set of DEPs is altered specifically in excitatory AD neurons, given our new platform to study this cell type in particular.

Another key concept gleaned from our analysis, at least under our conditions, is that the DEPs in the AD hiPSC‐neurons are mediating or triggering hyperexcitability, as opposed to hyperexcitability mediating the aberrant protein expression. For example, in Figure 4a the change in protein abundance in the mediation analysis with protein abundance as the mediator, AD mutant status as the treatment, and hyperelectrical activity (AUC) as the outcome suggests that aberrant protein expression in AD neurons drives the hyperelectrical activity phenotype. Furthermore, in Figure 4b, the lack of proteins that are up or downregulated in the mediation analysis with hyperelectrical activity (AUC) as the mediator, AD mutant status as the treatment, and protein abundance as the outcome is also consistent with the notion that aberrant protein expression in AD neurons drives the hyperelectrical activity, as opposed to the other way round. While our mediation analysis results make sense, our sample size was fairly small, suggesting that our analyses really show the potential of our technique for high‐resolution single‐cell proteomics exploring AD‐related molecular phenotypes for drug discovery purposes. Additionally, it is still possible that aberrant electrical activity could have an effect on certain proteins as a downstream effect or consequence, as synaptic activity is in many cases known to affect protein transcription.^[^ ^68^ ^]^ The fact that we do not see evidence for this in the current dataset could be because our experiment was not specifically designed to detect that. A related caveat is that local protein synthesis from transported RNAs occurs in neuronal dendrites near synapses^[^ ^69^ ^]^—therefore, the protein could be missing from the cell body where most of the proteins were aspirated from.

There are other limitations of our study. While we acknowledge that AD hiPSC‐neurons in culture would be expected to show only early manifestations of the disease, this can also be used to our advantage to explore changes in the proteome that may occur at early stages of AD. Moreover, the stress present in these culture systems may cause the neurons to age more quickly,^[^ ^70^ ^]^ and thus provide a reasonable model of neurodegenerative diseases of aging like AD. That said, using cultured cells like AD hiPSC‐neurons represents in vitro conditions, and thus informs on what is plausible rather than faithfully replicating in vivo conditions. Here, the approach allowed the assessment of human cells from a patient with the disease process and comparison to isogenic, gene‐corrected WT controls. The fact that several features of AD are faithfully reproduced in these AD hiPSC‐neurons such as the hyperelectrical phenotype and synaptic damage^[^ ^13^, ^14^ ^]^ gave us increased confidence that aspects of AD could be reproduced and studied, and this new technique can now be applied to other disease conditions as well.

## 4 Experimental Section

### hiPSC Lines

M146V/WT hiPSC lines bearing the PSEN1 M146V mutation (referred to as AD) and isogenic WT/WT control (referred to as WT) were used for this study and were obtained from the Marc Tessier–Lavigne lab, Rockefeller University/Stanford University and from the New York Stem Cell Institute. The details regarding these lines have been previously published.^[^ ^19^ ^]^

### hiPSC Maintenance and Differentiation

hiPSCs were differentiated to generate cerebrocortical neurons, as previously described.^[^ ^13^, ^14^, ^71^ ^]^ Briefly, feeder‐free hiPSCs were cultured using mTeSR1 medium (StemCell Technologies) on Matrigel. Differentiation of hiPSCs were induced by exposure to small molecules, 2 µm each of A83‐01 (Activin/Nodal inhibitor, Tocris), Dorsomorphin (bone morphogenetic protein inhibitor, Tocris), and PNU74654 (Wnt/β‐catenin inhibitor, Tocris) for 6 days in DMEM/F12 medium supplemented with 20% Knock Out Serum Replacement (Invitrogen, Carlsbad, CA). Cells were scraped manually to form PAX6+ neurospheres, which were maintained for ≈2 weeks in DMEM/F12 medium supplemented with N2 and B27 (Invitrogen) and 20 ng ml^−1^ of basic FGF. Thereafter, the neurospheres were seeded on poly‐l‐ornithine/laminin‐coated plates to form a monolayer of human neural progenitor cells (hNPCs) containing rosettes, which were expanded. For terminal differentiation, hNPCs were treated with 100 nm compound E (EMD Millipore, Temecula, CA) in BrainPhys medium (StemCell Technologies) for 48 h and then maintained in culture in BrainPhys medium. Cells at week 3 of terminal differentiation were switched to BrainPhys medium, and most experiments were conducted after 5–6 weeks of differentiation.

One NPC line per genotype was isolated and DNA sequencing to confirm the mutations was done using the primers supplied by the original lab.^[^ ^19^ ^]^ Routine quality controls include karyotyping after every ≈10 passages and frequent checks of cultures for possible mycoplasma contamination.

### Electrophysiology

Whole‐cell patch‐clamp recordings were performed as previously detailed.^[^ ^13^, ^14^, ^71^ ^]^ To reduce sample contamination by keratin or other human dermal proteins, investigators wore personal protective equipment (PPE) while performing experiments and handling samples. Patch pipettes had a resistance of 3–5 MΩ when filled with an internal solution composed of (in mm): K‐gluconate, 120; KCl, 5; MgCl_2_, 2; HEPES, 10; EGTA; 10; Mg‐ATP, 4; pH 7.4, and mOsm 290. The external solution was composed of Ca^2+^‐ and Mg^2+^‐free Hank's Balanced Salt Solution (HBSS; GIBCO, Gaithersburg, MD) to which CaCl_2_, 2 mm; HEPES, 10 mm; glycine, 20 µm; pH 7.4 were added. Patch pipettes were pulled from borosilicate glass capillaries (G150F‐3; Warner Instruments, Hamden, CT) using a micropipette puller (P2000; Sutter Instruments, Novato, CA). All recordings, including those for sodium‐potassium currents, evoked action potentials, etc., were performed using a Multiclamp 700B amplifier (Molecular Devices) at a data sampling rate of 10 kHz with a Digidata 1550B analog‐to‐digital convertor (Molecular Devices). Voltage‐clamp and current‐clamp protocols were applied using Clampex v.11 (Molecular Devices). Spontaneous excitatory postsynaptic currents (sEPSCs) were recorded in gap‐free mode at a holding potential of −70 mV. Under these conditions at 21 °C, the chloride ion reversal potential was ≈−70 mV; hence, inward synaptic currents recorded at −70 mV represented excitatory responses. Preliminary analysis and offline filtering at 500 Hz were achieved using Clampfit v.11 (Molecular Devices). Area‐under‐the‐curve (AUC) analysis of sEPSCs was performed on 20 s epochs of uninterrupted recording.

### Mass Spectrometry

Immediately after recording, the AD or WT hiPSC‐neuron was gently aspirated into the patch pipette, and then the whole neuron was lifted up off of the dish with the pipette. Subsequently, the cell was expelled by positive pressure and slightly breaking the tip of the pipette into a vial containing trypsin. The cellular contents were digested with trypsin (10 ng µl^−1^) in acetic acid at 60 °C for 1 h on a heating block.

The samples were subsequently analyzed on an Orbitrap Eclipse Tribrid mass spectrometer (Thermo). Samples were injected directly onto a 25 cm, 100 µm ID column packed with BEH 1.7 µm C18 resin (Waters). Samples were separated at a flow rate of 300 nL min^−1^ on an EasynLC 1200 (Thermo). Buffer A and B were 0.1% formic acid in water and 90% acetonitrile, respectively. A gradient of 1–25% B over 100 min, increased to 40% B over 20 min, increased to 90% B over 10 min, and then held at 90% B for a 10 min was used for a 140 min total run time.

Peptides were eluted directly from the tip of the column and nanosprayed directly into the mass spectrometer by application of 2.5 kV voltage at the back of the column. The Eclipse was operated in a data dependent mode. Full MS1 scans were collected in the Orbitrap at 120 K resolution. The cycle time was set to 3 s, and within these 3 s the most abundant ions per scan were selected for HCD MS/MS at 60 K detection in the Orbitrap. Monoisotopic precursor selection was enabled, and dynamic exclusion was used with exclusion duration of 50 s.

### Data Processing and Statistics

In total, 118 cells (from the 140 recorded) could be fully analyzed using this workflow, 57 AD and 61 WT hiPSC‐neurons. The protein false discovery rate (FDR) in the search was ≤1% of the protein level. Accordingly, any cell which had less than 100 total proteins identified was removed from the analysis as it was considered a “failed” collection. Moreover, any protein that was identified in less than 5 cells was removed as it was considered to be either a false hit or too low in abundance to measure reliably. There were 3 biological replicates in these experiments, representing 3 separate growth/plating dates of hiPSCs performed several months apart and on different clones. Since the LC‐MS was done one shot per cell, there are no so‐called technical replicates, but the fact that 140 cells were analyzed in this manner gave the study sufficient power for statistical comparison.

The average protein count after peptide mapping though MaxQuant was ≈520 per cell for the AD line, and 470 for the WT line. In order for these proteins to be carried over for downstream analyses, they were required to be found in at least 5 cells. When this first‐pass filter was applied to the 2251 proteins detected, 1382 different proteins were found across both genotypes. Several of which, 37 in total, were observed to be differentially abundant dependent on genotype. The abundances of these proteins, measured here as FC, were in terms of AD genotype relative to WT. On standard Volcano plots for proteins analyzed, log (probability) versus log (protein magnitude) was graphed, with cut‐offs for significance representing ≥ 1.5x FC in mean expression value by label‐free quantification (AD proteins relative to WT) with *P* < 0.05.

### Bioinformatics Analysis—Differential Protein Abundance Analysis

To identify proteins that are differentially expressed between AD mutant‐bearing and non‐AD mutant bearing cells, standard linear regression analysis was used^[^ ^72^ ^]^ with AD mutation status coded as a dummy variable *t*, with *t* = 0 or 1, depending on whether a cell did not harbor a mutation *t* = 0, or did *t* = 1, respectively, as an independent variable and protein abundance as the dependent variable. The linear regression was also used for exploring the relationships between protein abundance levels and electrophysiology phenotypes with the electrophysiology variables taken as a dependent variable and protein abundances as the independent variable. The interaction terms (e.g., mutation status x protein abundance level) were included in these models as well.

### Mediation Analysis

Regression‐based mediation analysis methods were used to find proteins that appear to be affected by the AD mutations that, in turn, affect the neurophysiological measures across the cells. In addition, the opposite hypothesis – that the neurophysiological phenotypes are affected by the AD mutations and they, in turn, affect protein abundances as a consequence or secondary effect – was also tested. The techniques pursued for the regression‐based mediation analysis are well‐known and used often in a wide variety of contexts.^[^ ^73^, ^74^ ^]^

## Conflict of Interest

The authors declare no conflict of interest.

## Author Contributions

S.G. and J.K.D. contributed equally to this work. M.A., S.G., J.K.D., J.R.Y. III, and S.A.L. performed conceptualization. M.A., S.G., M.T., and J.K.D. performed methodology and investigation. H.S., S.G., M.S., N.B., and N.J.S. performed bioinformatics and visualization. S.A.L., M.A., J.R.Y. III, and N.J.S. performed funding acquisition. S.A.L. wrote the original draft. S.G., J.R.Y. III, J.K.D., N.J.S., and S.A.L. reviewed and edited the author manuscript.

## Supporting information

Supporting Information

Supplemental Table 1

Supplemental Table 2

Supplemental Table 3

Supplemental Table 4

Supplemental Table 5

## Data Availability

The data that support the findings of this study are available in the supplementary material of this article.
