# Supplementary material for: Single‐Cell Patch‐Clamp/Proteomics of Human Alzheimer's Disease iPSC‐Derived Excitatory Neurons Versus Isogenic Wild‐Type Controls Suggests Novel Causation and Therapeutic Targets
Source: Adv Sci (Weinh). 2024 May 21;11(29):2400545. doi: 10.1002/advs.202400545 (PMC11304297; doi:10.1002/advs.202400545)
Supplement: Supplementary file 1 — Supporting Information [file ADVS-11-2400545-s001.docx]

**Supporting Information**

**Title: Single cell patch-clamp/proteomics of human Alzheimer’s disease iPSC-derived excitatory neurons vs. isogenic wild-type controls suggests novel causation and therapeutic targets**

Swagata Ghatak^1,2,5^, Jolene K. Diedrich^2,5^, Maria Talantova^1,2^, Nivedita Bhadra^3^, Henry Scott^1,2^, Meetal Sharma^1,2^, Matthew Albertolle^1,2^, Nicholas J. Schork^3^, John R. Yates III^2^ &

Stuart A. Lipton^1,2,4^

**Supplementary Figure S1, Tables S1-5.**

**
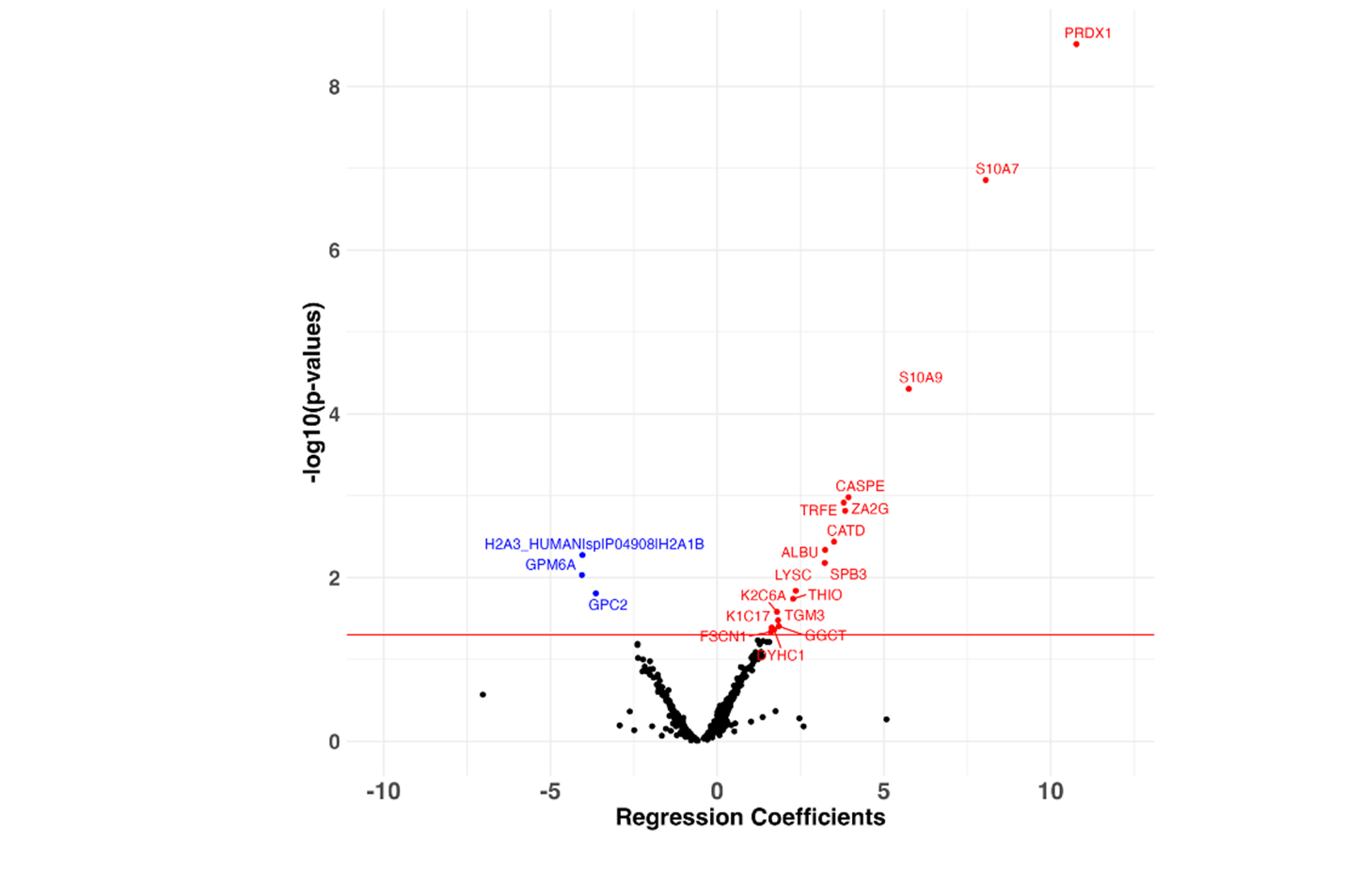
**

**Fig. S1 (data from Model 2 in Table S5).** Volcano plot of linear regression model results with log(AUC) taken as the dependent variable and protein abundance taken as the independent variable. By this analysis, 16 proteins were upregulated, and 3 proteins were downregulated in AD hiPSC-neurons compared to isogenic WT; redline indicates protein FC ≥ 1.5 and *P* < 0.05.

**Table S1.** List of all proteins and their label free quantification (LFQ) intensity from MS that were detected in the 118 hiPSC-neuron dataset (AD = 57, WT = 61). See separate EXCEL file.

**Table S2.** All proteins detected and electrophysiological data for the 57 hiPSC-neurons (AD = 29, WT = 27) used for analysis. Note that in this 57-cell subset of the data, a total of 1,390 proteins were detected following the criteria that each protein had to be present in at least five of the cells, and each cell had to contain at least 100 of the detected proteins. See separate EXCEL file.

**Table S3.** Functional enrichment analysis results using g:Profiler software for the 118 hiPSC-neuron dataset (AD = 57, WT = 61). See separate EXCEL file.

**Table S4**. LFQ intensity from MS data for each protein in the 57 (AD = 29, WT = 28) hiPSC neurons analyzed that also had adequate electrophysiological data for analysis. See separate EXCEL file.

**Table S5**. List of DEPs in the AD vs. isogenic WT hiPSC-neurons used in the linear regression and mediation models presented in the text. See separate EXCEL file.
